# Characterization of wheat *Bell1*-type homeobox genes in floral organs of alloplasmic lines with *Aegilops crassa *cytoplasm

**DOI:** 10.1186/1471-2229-11-2

**Published:** 2011-01-04

**Authors:** Kota Mizumoto, Hitoshi Hatano, Chizuru Hirabayashi, Koji Murai, Shigeo Takumi

**Affiliations:** 1 Graduate School of Agricultural Science, Kobe University, Nada-ku, Kobe 657-8501, Japan; 2 Department of Bioscience, Fukui Prefectural University, Yoshida-gun, Fukui 910-1195, Japan

## Abstract

**Background:**

Alloplasmic wheat lines with *Aegilops crassa *cytoplasm often show homeotic conversion of stamens into pistils under long-day conditions. In the pistillody-exhibiting florets, an ectopic ovule is formed within the transformed stamens, and female sterility is also observed because of abnormal integument development.

**Results:**

In this study, four wheat *Bell1*-like homeobox (*BLH*) genes were isolated and named *WBLH1 *to *WBLH4*. *WBLH1*/*WBLH3*/*WBLH4 *expression was observed in the basal boundary region of the ovary in both normal pistils and transformed stamens. *WBLH2 *was also strongly expressed in integuments not only of normal ovules in pistils but also of the ectopic ovules in transformed stamens, and the *WBLH2 *expression pattern in the sterile pistils seemed to be identical to that in normal ovules of fertile pistils. In addition, WBLH1 and WBLH3 showed interactions with the three wheat KNOX proteins through the BEL domain. WBLH2, however, formed a complex with wheat KNOTTED1 and ROUGH SHEATH1 orthologs through SKY and BEL domains, but not with a wheat LIGULELESS4 ortholog.

**Conclusions:**

Expression of the four *WBLH *genes is evident in reproductive organs including pistils and transformed stamens and is independent from female sterility in alloplasmic wheat lines with *Ae. crassa *cytoplasm. KNOX-BLH interaction was conserved among various plant species, indicating the significance of KNOX-BLH complex formation in wheat developmental processes. The functional features of *WBLH2 *are likely to be distinct from other *BLH *gene functions in wheat development.

## Background

Alien cytoplasm largely alters gene expression profiles, affecting growth and organogenesis. Nuclear-cytoplasm incompatibility results in abnormal growth phenotypes in higher plants [1,2]. Recurrent backcrossing has been commonly used for production of nuclear-cytoplasmic substitution plants called alloplasmic lines, in which the cytoplasmic genomes are replaced by ones from a related species [3]. Cytoplasmic male sterility is a major phenomenon among the abnormal phenotypes of the alloplasmic lines [1,4]. In many cases of cytoplasmic male sterility, nuclear-cytoplasm incompatibility induces abortion of pollen. Homeotic transformation of stamens into pistil-like structures is sometimes observed in alloplasmic lines of carrot, *Brassica napus*, tobacco and wheat [5-9], a phenomenon called pistillody.

**Figure 1:**
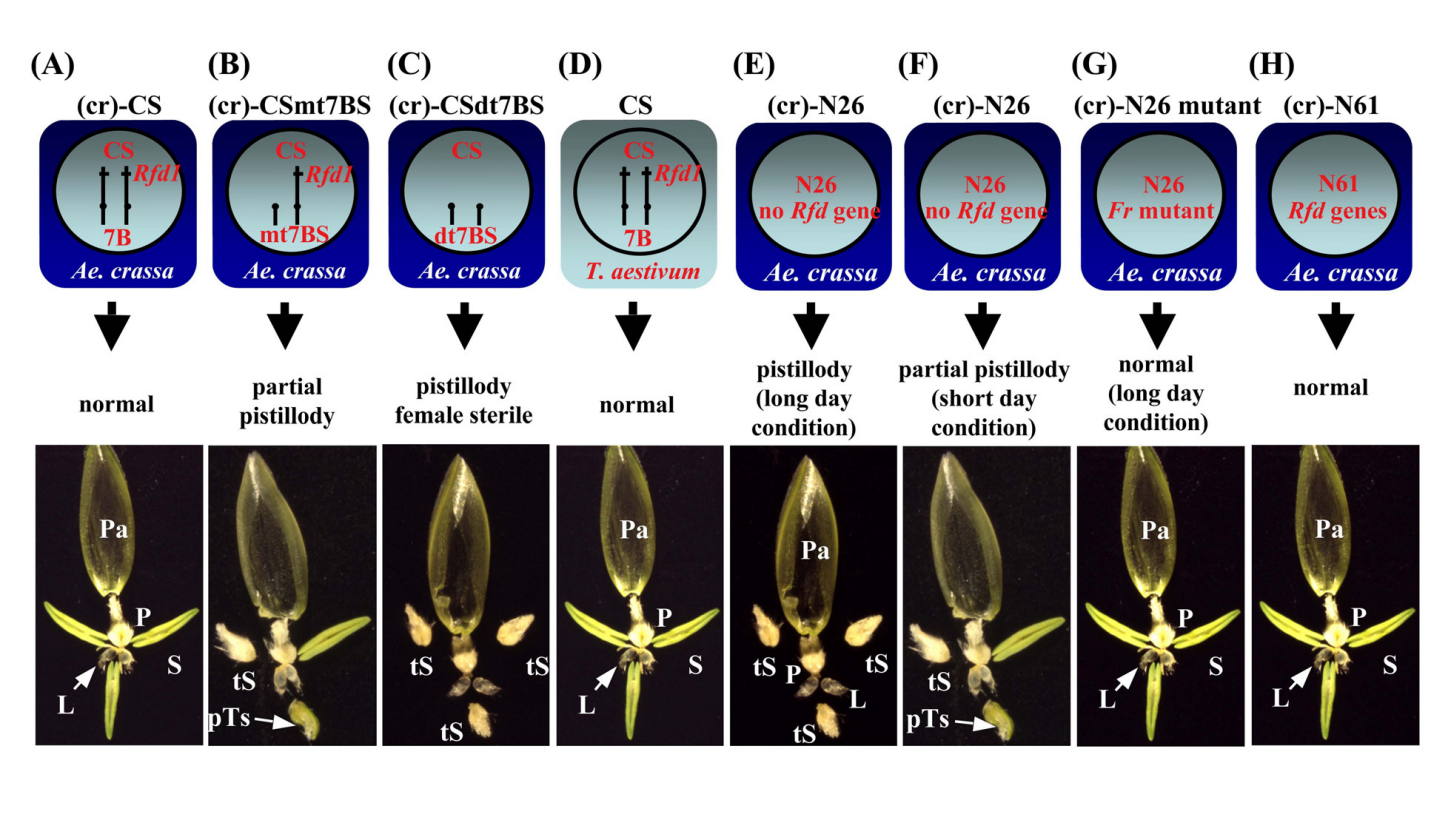
**Photoperiod-sensitive cytoplasmic male sterility in alloplasmic wheat with *Ae. crassa *cytoplasm**. Genetic characteristics of nuclear and cytoplasmic types and floral organ morphology without the lemma in the alloplasmic lines are schematically represented. (A) (*cr*)-CS has normal stamens because of the fertility restorer gene *Rfd1 *against the *Ae. crassa *cytoplasm on the long arm of chromosome 7B. (B) (*cr*)-CS monotelodisomic 7BS, which has *Rfd1 *in a hemizygous state, shows partial pistillody (arrow) and reduced female fertility. (C) (cr)-CS ditelosomic 7BS, which lacks both of the long arms pair of chromosome 7B and therefore lacks *Rfd1*, shows complete pistillody and female sterility, independent of day length. Floral organ morphology of CS ditelosomic 7BS was indistinguishable from that of CS. (D) Euplasmic CS. (E) (cr)-N26 shows homeotic transformation of stamens into pistils (pistillody) only under long-day conditions. (F) (cr)-N26 occasionally shows partial pistillody (arrow) and reduced female fertility under short-day conditions. (G) An EMS-induced mutant of (cr)-N26 shows normal floral phenotype [36]. This mutant fails to measure day length. (H) (cr)-N61, which contains multiple loci for *Rfd *genes, shows normal floral phenotype. Pa, palea; P, pistil; S, stamen; L, lodicule; pTs, partially transformed stamen; Ts, transformed stamen.

Cytoplasm of a wild wheat relative, *Aegilops crassa*, homeotically affects floral organ development and induces pistillody in some alloplasmic common wheat lines lacking fertility restorer genes against *Ae. crassa *cytoplasm (Figure 1) [10,11]. The mitochondrial *orf260 *gene in the *Ae. crassa *cytoplasm might be associated with induction of the floral homeotic change [12]. An alloplasmic line of the wheat cultivar Norin 26 (N26) with *Ae. crassa *cytoplasm [(cr)-N26] exhibits male sterility under long-day conditions (> 15 h light period) due to pistillody (Figure 1E, F), a phenomenon termed photoperiod-sensitive cytoplasmic male sterility [10]. However, an alloplasmic line of the wheat cultivar Chinese Spring (CS) does not because of a fertility restorer gene *Rfd1*, located on the long arm of chromosome 7B (Figure 1A) [13]. An alloplasmic line of CS (ditelosomic 7BS) with *Ae. crassa *cytoplasm [(cr)-CSdt7BS] lacking both long arms of the homologous chromosome 7B shows pistillody unrelated to day length (Figure 1C), but CS ditelosomic 7BS with the intrinsic cytoplasm (CSdt7BS) forms normal stamens (Figure 1D) [6].

The *Ae. crassa *cytoplasm alters expression patterns of wheat class B MADS-box genes at the floral meristem of (cr)-CSdt7BS [6]. Primordia of the pistil-like stamens lack expression of wheat *APETALA3 *(*AP3*) and *PISTILLATA *(*PI*) orthologs such as *WAP3*, *WPI-1 *and *WPI-2 *[14], whereas two wheat class C MADS-box genes, wheat *AGAMOUS *paralogs (*WAG-1 *and *WAG-2*), are ectopically expressed at the pistil-like stamens [15,16]. Despite initiation of ovule formation in these transformed stamens, ectopic ovules incompletely develop, and the transformed stamens are sterile. In addition, pistils are also sterile in the (cr)-CSdt7BS alloplasmic line, and female sterility is due to abnormal ovule development [6]. Both the ovules and ectopic ovules fail to form an inner epidermis and integuments in the chalaza region. This incomplete development of the ovules strongly suggests alteration of gene expression patterns essential to normal ovule formation in pistils and pistil-like stamens of the (cr)-CSdt7BS alloplasmic line. However, there is little information about gene expression associated with wheat ovule development.

Following determination of carpel identity via *AGAMOUS *(*AG*), a class C MADS-box gene acting to specify stamen and carpel development in *Arabidopsis *[17], ovule primordia are initiated within pistils. The initiation of ovule primordium formation is caused by expression of class D MADS-box genes such as *Arabidopsis SEEDSTICK *(*STK*) and petunia *floral binding protein 7 *(*FBP7*) and *FBP11 *[18-20]. *ARABIDOPSIS BSISTER *(*ABS*) and *FBP24 *from petunia are members of the B_sister _subfamily, and are necessary to determine the identity of the endothelial layer within the inner integument of the ovule [21,22]. Our previous study using wheat alloplasmic lines with *Ae. crassa *cytoplasm demonstrated that alteration of class B and C MADS-box genes was connected with continuous transcript accumulation of class D and B_sister _MADS-box genes, *WSTK *and *WBsis*, respectively, in primordia of ectopic ovules within the pistil-like stamens [23]. Moreover, the *WBsis *transcript is not restricted to the endothelium and appears at the nucellus in ectopic ovules in (cr)-CSdt7BS. *Arabidopsis AINTEGUMENTA *(*ANT*) plays an important role in ovule development along the proximal-distal axis [24]. *ant *mutants fail to both initiate and elongate the integument [25,26]. The aberrant ovule formation is at least partly associated with the weak expression of a wheat *ANT *homolog *WANT-1 *around ovule primordia in (cr)-CSdt7BS [16]. Therefore, alteration of gene expression after initiation of ovule primordia results in abnormal ovule development in alloplasmic wheat with *Ae. crassa *cytoplasm.

*Arabidopsis *homeodomain protein BELL1 (BEL1) is also required for ovule morphogenesis [27], suggesting that wheat *BEL1 *homologs may be associated with ovule development. *BEL1 *acts to specify integument identity by controlling *AG *activity [28]. *BEL1 *encodes a TALE homeodomain transcription factor distantly related to the PBX family [27]. Plant *BEL1*-like homeobox (*BLH*) genes form a small gene family functioning in various developmental aspects such as seed shattering in rice [29] and leaf shape establishment in *Arabidopsis *[30]. For many of the *BLH *functions, molecular interaction with KNOTTED1-type homeodomain (KNOX) proteins is required. Barley *JuBel1 *and *JuBel2 *were originally isolated as interaction partners of the KNOX protein BKN3 [31]. *Arabidopsis BELLRINGER *(syn. *PENNYWISE*) functions with *KNOX *genes in early patterning of inflorescence architecture and in maintenance of the inflorescence meristem [32,33]. The BEL domain of BLH directly interacts with the MEINOX domain of KNOX, and the interaction is evolutionarily conserved between animals and plants [34,35].

To elucidate molecular mechanisms of the abnormal ovule development in alloplasmic wheat with *Ae. crassa *cytoplasm, we first isolated wheat cDNA clones for the *BLH *homologs, and then studied their differential expression patterns and interaction with wheat KNOX proteins. Association of the *BLH *homologs with pistillody and female sterility in (cr)-CSdt7BS is discussed based on the results.

## Methods

### Plant materials

Three common wheat (*Triticum aestivum *L., genome constitution AABBDD) cultivars, Chinese Spring (CS), Norin26 (N26) and Norin61 (N61), and a ditelosomic 7BS line of CS (CSdt7BS) were used in this study. Euplasmic lines of each with their intrinsic cytoplasm develop normal fertile reproductive organs. Alloplasmic lines with the cytoplasm of a wild relative, *Aegilops crassa*, used in the study were respectively abbreviated as (cr)-CS, (cr)-N26, (cr)-N61 and (cr)-CSdt7BS. The (cr)-N26 shows pistillody when grown under long-day conditions (Figure 1E), and exhibits partial pistillody under short-day conditions, implying that N26 might contain a fertility restorer (*Rf*) gene functioning under short-day conditions (Figure 1F) [10]. Both (cr)-CS and (cr)-N61 show normal male fertility because of their possession of *Rf *gene(s) against the *Ae. crassa *cytoplasm in their nuclear genome (Figure 1A, H) [11]. CS has a single dominant *Rf *gene, *Rfd1*, on the long arm of chromosome 7B (7BL) and N61 has multiple *Rf *genes. A CS monotelodisomic line of chromosome 7BS with the *Ae. crassa *cytoplasm [(cr)-CSmt7BS] is hemizygous for *Rfd1 *and partially fertile (Figure 1B). (cr)-CSdt7BS, which was generated from a cross of (cr)-CSmt7BS with CSdt7BS, shows not only pistillody but also female sterility, independently of day length (Figure 1C) [6]. An *Fr *mutant of (cr)-N26 obtained after EMS mutagenesis of (cr)-N26 shows no pistillody even under long-day conditions (Figure 1G) [36], and was used in the expression analysis.

Three accessions of ancestral diploid species, *Triticum urartu *(AA), *Aegilops speltoides *(SS) and *Aegilops tauschii *(DD), and the tetraploid wheat (*Triticum durum*, AABB) cultivar Langdon were used in DNA gel blot analysis. A nulli-tetrasomic series of CS produced by Sears [37] was used for chromosome assignment of the isolated wheat cDNAs. Each line of the nulli-tetrasomic series lacks a given pair of homoeologous A, B or D genome chromosomes (the nullisomic condition) that have been replaced by the corresponding homoeologous chromosome pair (the tetrasomic condition).

### Cloning and sequencing of wheat *BELL1 *homologs

Degenerate primers, 5'-CGA(A/G)CACTTCCT(A/G/C/T)CACCCGT-3' and 5'-AC(C/G)C(G/T)(C/G/T)GCGTTGAT(A/G)AACCA-3', were designed and used for amplification of the BEL domain regions in RNA from pistils of CS and (cr)-CSdt7BS. The reverse transcription (RT)-PCR products were cloned into the pGEM-T Easy vector (Promega, Madison, WI, USA) and nucleotide sequences were determined by an automated fluorescent DyeDeoxy terminator cycle sequencing system using an ABI PRISM 310 genetic analyzer (Applied Biosystems, Foster City, CA, USA). Nucleotide sequences of the isolated cDNA fragments were analyzed by DNASIS software (Hitachi, Tokyo, Japan) and the sequence was searched for homology using the BLAST algorithm [38].

The cDNA fragments were used to search wheat expression sequence tag (EST) clones, and the identified EST clone TaLr1107F03R contained the complete open reading frame (ORF). To identify other BLH cDNA clones, first-strand cDNA was synthesized using total RNA from pistils of CS and (cr)-CSdt7BS, and 5' and 3' RACE-PCR was performed with a SMART-RACE PCR kit (BD Bioscience Clontech, Tokyo, Japan) according to the manufacturer's procedure. Gene-specific primers for RACE-PCR were designed based on the nucleotide sequences of the degenerate primer-amplified RT-PCR products. For 5' and 3' RACE-PCR, the following primers were respectively used: 5'-TCCTCGCCGCCAGCATGTCCTTCTCGT-3' and 5'-AAGTCCGTCGCCGTGCTCAAAGCCTGG-3' for *WBLH2*, 5'-TTTGAGACCTGATTCCTCGTTAAGCCTG-3' and 5'-CAGCAAGGTGGCCGCCGGGAAAGACAG-3' for *WBLH3*, and 5'-GGCCAGCATTTGCTTGTCGCCATCGGTA-3' and 5'-GCGCAACACCAGCAAGATGCCGGTCAA-3' for *WBLH4*. A cDNA fragment containing the complete ORF was amplified with the following primer sets: 5'-CCGACGACGACGATGATGAC-3' and 5'-CCTTAGCCCCCCCAAGAATA-3' for *WBLH1*, 5'-CGGTGCTTTCTCTCTCCATG-3' and 5'-TTCCTAGCCGACGACGTCTT-3' for *WBLH2*, and 5'-CCTCCCTCTCTCTCCCCCTT-3' and 5'-TGATCCCAGCAATGGAGCAA-3' for *WBLH3*. The nucleotide sequences of the isolated cDNA clones were analyzed as mentioned above, and a phylogenetic tree was constructed by GENETYX-MAC version 12.00 software (Whitehead Institute for Biomedical Research, Cambridge MA) based on Nei's genetic distance. The phylogenetic tree was constructed according to the unweighted pair group method with arithmetic mean (UPGMA) method [39].

### Southern blot and RT-PCR analyses

**Figure 2:**
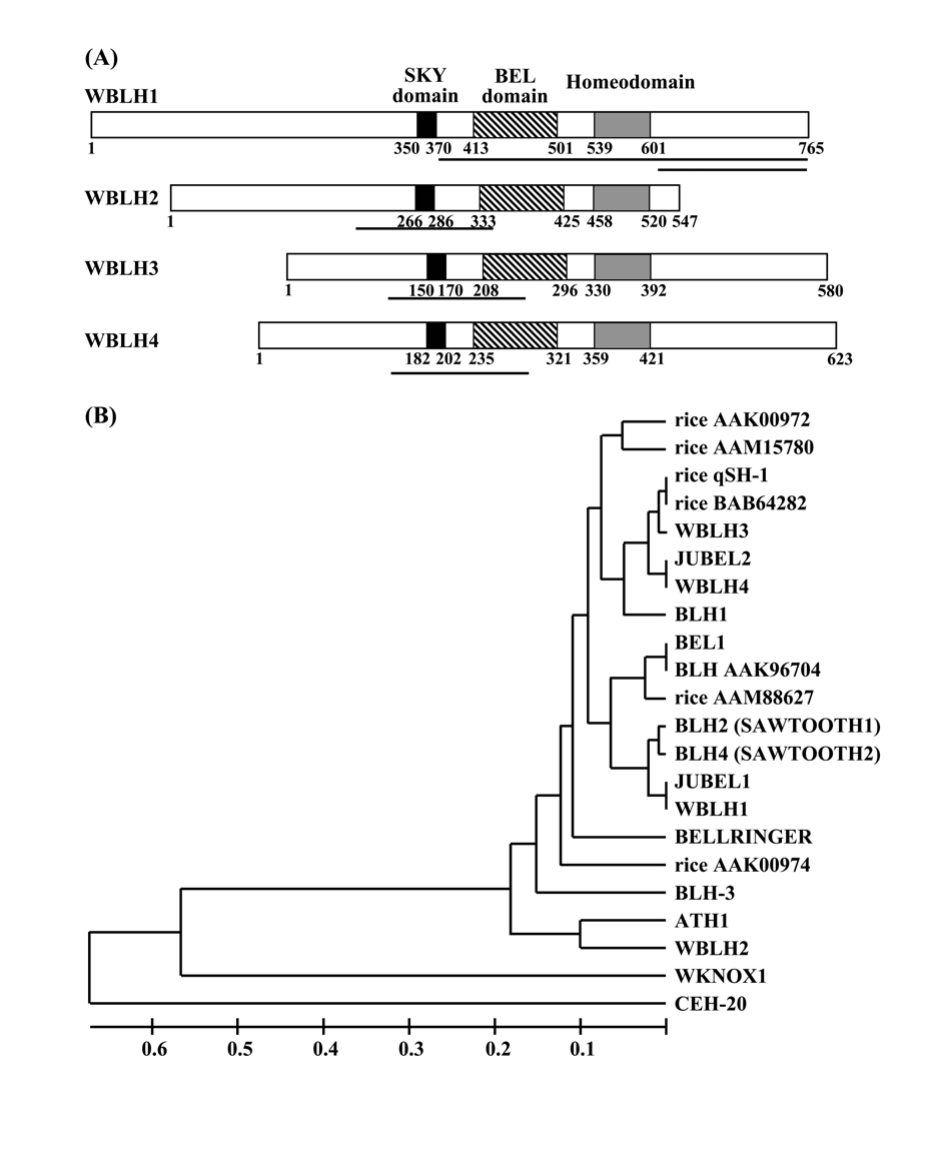
**Four *BEL1*-type homeobox genes in common wheat**. (A) Schematic representation of structures of four putative WBLH proteins. Probe positions used in Southern blot analyses are underlined. Numbers indicate the amino acid positions of individual domains. (B) A phylogenetic tree based on the homeodomain sequences of BLH proteins. In addition to WBLH1 to WBLH4, six rice BLH, eight *Arabidopsis *BLH and barley JUBEL1 and JUBEL2 proteins were included. Homeodomain sequences of WKNOX1, which is a member of the wheat class 1 KNOX protein family, and *Caenorhabditis elegans *CEH-20, which is a homolog of PBX protein, were used as outgroups. The phylogenetic tree was constructed according to the UPGMA method.

For genomic Southern blot analysis, total DNA extracted from CS, Langdon and diploid wheat was digested with the restriction enzyme *Hin*dIII. Total DNA extracted from the nulli-tetrasomics was also digested with *Hin*dIII, *Dra*I and *Bam*HI. The digested DNA was fractionated by electrophoresis through a 0.8% agarose gel, transferred to Hybond N^+ ^nylon membrane (GE Healthcare, Piscataway, NJ, USA) and hybridized with ^32^P-labeled partial *BLH *cDNA fragments as a probe (Figure 2). Probe labeling, hybridization, washing and autoradiography were performed according to Takumi et al. [40].

Total RNA was extracted by guanidine thiocyanate from various tissues at the vegetative and reproductive phases in the euplasmic and alloplasmic wheat lines. Accumulation of the four *BLH *and three *KNOX *transcripts was detected by RT-PCR amplification as previously reported [41]. RT-PCR was conducted with the following gene-specific primer sets: 5'-TCAACCGACAGCAGCAGCAG-3' and 5'-CCGAACCCCATCACCGAGTC-3' for *WBLH1*, 5'-GTGCCCAGTCTTCCTCGGTC-3' and 5'-TCCATCCACCTCCCGCCGTC-3' for *WBLH2*, 5'-CGCTGTCCTCGTCCTCCTCG-3' and 5'-GGAGAGCGATGGAGGCAAAG-3' for *WBLH3*, and 5'-CCCTCTCCTCCGCCTCGTCC-3' and 5'-CGGGGCGGCGTTGCTGAACC-3' for *WBLH4*. Primers for amplification of *Wknox1*, *WRS1 *and *WLG4 *(AB465042) transcripts are described in our previous studies [42-44]. The ubiquitin (*Ubi*) and actin (*Act*) genes were used as internal controls [41,45]. The PCR-amplified products were separated by electrophoresis through a 1.5% agarose gel and stained with ethidium bromide. RT-PCR analysis was performed at the exponential range of amplification, and the entire experiment was conducted twice. Two technical replicates were performed for each biological replicate.

Quantitative RT-PCR was performed using a TaKaRa Thermal Cycler Dice Real Time System (TaKaRa Bio, Ohtsu, Japan) and gene-specific primer sets. As an endogenous control, the wheat *Act *gene was used. The rate of amplification was monitored using THUNDERBIRD SYBR pPCR Mix (Toyobo, Osaka, Japan) according to the manufacturer's protocol. Results were obtained as 2^-^Δ^Ct ^values, where Ct is the number of PCR cycles required to reach the log phase of amplification for the examined genes minus the same measure for *Act*, and were represented as values relative to the transcript levels in pistils of CS, N61 or CSdt7BS.

### mRNA in situ hybridization

Pistils and young spikes at the stage of floral organ development in the lines CSdt7BS and (*cr*)-CSdt7BS were fixed in 3.7% *p*-formaldehyde, 5% acetic acid at 4°C overnight. The fixed tissues were embedded in Paraplast medium (Oxford Labware, St. Louis, MO, USA) after dehydration with ethanol and infiltration with xylene. The embedded samples were sliced into 8-μm sections and dried overnight onto slides coated with VECTABOND Reagent (Vector Laboratories, Burlingame, CA, USA). Tissue sections were deparaffinized with xylene and hydrated through an ethanol series, then dehydrated through an ethanol series after treatment with proteinase K and triethanolamine. Hybridization of the four *BLH *mRNAs with digoxigenin-labeled probe produced from the coding regions was performed overnight at 52°C. The RNA probes were directly synthesized with T3 and T7 RNA polymerase (Toyobo) from the isolated cDNA clone. After hybridization, the sections were washed and treated with RNase. Immunological detection of the hybridized probe was according to Morimoto et al.[42].

### Yeast two-hybrid assay

A HybriZAP-2.1 two-hybrid undigested vector kit (Stratagene, La Jolla, CA, USA) was used to study protein-protein interaction between BLH and KNOX. The entire ORF sequences of wheat *BLH *and *KNOX *cDNA fragments were amplified with the following primer sets containing either an *Eco*RI, *Bam*HI, *Sal*I or *Xba*I linker: 5'-CCGAATTCATGGGAATAGCGGCGCCACC-3' and 5'-CCGTCGACTCAACAACCATTGTAGTCTC-3' for *WBLH1*, 5'-GGGGATCCATGTCTAGCAATCCATCCTA-3' and 5'-GGTCTAGATTCCTAGCCGACGACGTCTT-3' for *WBLH2*, 5'-GGGGATCCGCCGCCGCCATGTCATCGG-3' and 5'-GGGGATCCGTCGACGATCACCCAACGAGTCAT-3' for *WBLH3*, 5'-CCGAATTCATGGAGGAGATCGGCCACCA-3' and 5'-CCCCCGGGCTAGCCGAACCTGTAGAGCC-3' for *Wknox1*, 5'-GGGAATTCATGGAGAAGTTCCCTAAT-3' and 5'-GGGTCGACTGGAGAAAGGGAGAGAGG-3' for *WRS1*, and 5'-GGGAATTCATGGAGGATCTGTACAGCA-3' and 5'-GGGTCGACAGCAATCATCCATCCATCT-3' for *WLG4*. The PCR products were digested with *Eco*RI and *Sal*I and cloned into the *Eco*RI/*Sal*I sites of pAD-GAL4-2.1 and pBD-GAL4 Cam vectors, resulting in pAD-WBLH1, pAD-WBLH2, pAD-WBLH3, pBD-WKNOX1, pBD-WRS1, pBD-WLG4 and pBD-WBLH1. Deletion derivatives of pAD-WBLH1, pAD-WBLH2 and pBD-WBLH1 were generated using internal restriction enzyme recognition sites and internal primers with the linker sequence. pAD-WT and pBD-WT, containing the wild-type fragment C of lambda cI, were used as controls according to the manufacturer's protocol (Stratagene). These pAD and pBD constructs were introduced into yeast strain YRG-2 (Stratagene). The interaction was assessed on SD medium (Q-BIOgene, Irvine, CA, USA) without leucine (Leu), tryptophan (Trp) or histidine (His) and containing 3 mM 3-amino-1,2,4-triazole.

## Results

### Cloning of wheat *Bell1*-like homeobox genes

The CS line ditelosomic 7BS lacking *Rfd1 *with normal cytoplasm (CSdt7BS) forms normal stamens, whereas the alloplasmic line of CSdt7BS with *Ae. crassa *cytoplasm ((cr)-CSdt7BS) showed pistillody in all florets (Figure 1). To identify wheat *BLH *homologs associated with pistillody, we designed degenerate primers based on the nucleotide sequences of rice and barley *BLH *homologs, and amplified the 108-bp regions encoding the conserved BELL1-type homeodomains using total RNA from pistils of CS and (cr)-CSdt7BS. In total, 63 of the RT-PCR products were sequenced and classified into seven groups based on their nucleotide sequences (Additional file 1), indicating that at least seven members of the *BLH *gene family were identified in the wheat genome. Four of the identified *BLH *members, groups 1, 2, 3 and 7, appeared favored by either CS or (cr)-CSdt7BS.

In the wheat EST database of the Cereal Research Centre, Agriculture and Agri-Food Canada, one EST clone showed high homology to barley *JuBel1 *[31], and the cDNA clone TaLr1107F03R contained an entire ORF. The wheat *JuBel1 *homolog corresponding to the group 3 RT-PCR products was named *WBLH1*. Two additional cDNA sequences for *WBLH1 *were isolated from CS. The three *WBLH1 *cDNAs contained single ORFs encoding 767, 765 and 771 amino acid residues, and were respectively designated *WBLH1-1*, *WBLH1-2*, and *WBLH1-3*. To isolate full-length cDNA clones for the group 1, 2 and 7 RT-PCR products, 5'- and 3'-RACE-PCR were conducted with gene-specific primers designed for the homeobox regions. Based on the nucleotide sequences of the RACE-PCR products, three cDNA-specific primer sets were designed, and the cDNA clones identified were named *WBLH2*, *WBLH3 *and *WBLH4*. A *WBLH2 *cDNA clone encoding 547 amino acid residues was obtained, and the cDNA sequence included an 18-bp deletion in the ORF compared with the RACE-PCR product. The *WBLH3 *and *WBLH4 *cDNA clones contained an entire ORF encoding 580 and 623 amino acids, respectively. The isolated cDNA sequences were deposited in the DDBJ database under the accession numbers AB546641-AB546647.

Putative amino acid sequences of the four wheat BLH proteins contained three conserved domains, SKY, BEL and homeodomains (Figure 2A). Based on the homeodomain sequences of four WBLH and related proteins, a phylogenetic tree was constructed (Figure 2B). The amino acid sequence of WBLH1-1 showed the highest similarity (90.2% identity) to that of barley JuBEL1, and JuBEL2 was closely related (78.7% identity) to WBLH4. WBLH3 showed high similarity to rice qSH1, a major quantitative trait locus controlling seed shattering through formation of an abscission layer [29]. The homeodomain of WBLH2 was highly similar to that of *Arabidopsis *light-induced ATH1 [46] and distantly divergent from other BEL1-related proteins. In all three conserved domains, WBLH1 showed the highest similarity to *Arabidopsis *BEL1 of the four WBLH proteins. Among these WBLH proteins, homeodomain sequences were highly conserved, while the SKY and BEL domains were relatively more diverged (Additional file 2). In particular, the SKY and BEL domains of WBLH2 were distinct from those of the other three WBLH proteins.

To study the copy number of *WBLH1 *in the wheat genome, Southern blots were analyzed using total DNA isolated from diploid, tetraploid and hexaploid wheat. Southern blots using the shorter cDNA fragment as a probe (Figure 2A) showed low copy numbers of *WBLH1 *in tetraploid and hexaploid wheat genomes, and a single major and a few minor bands for *WBLH1 *in the A, S and D diploid genomes (Additional file 3). To assign the *WBLH1 *homologous loci to wheat chromosomes, aneuploid analysis was performed using a series of nulli-tetrasomic lines. Southern blots using the longer cDNA fragment as a probe (Figure 2A) showed that *WBLH1*-homologous bands could be assigned to chromosomes 2B, 4A, 4D, 5A, 5B and 5D (Additional file 3), meaning that the three isolated *WBLH1 *cDNAs were not necessarily homoeologous. Two major bands of *WBLH2 *were observed in the A, S and D diploid genomes (data not shown), and the *WBLH2*-specific bands were absent only in the nulli-tetrasomic lines of homoeologous group 7 chromosomes and chromosomes 1A, 2A and 2D (Additional file 3). *WBLH2 *and its homologous loci were assigned to the six chromosomes in common wheat. Similarly, a single major band for *WBLH3 *and *WBLH4 *was detected in the A, S and D diploid genomes (data not shown). The *WBLH3*- and *WBLH4*-specific bands were absent in the nulli-tetrasomic lines of homoeologous group 1 and 3 chromosomes, respectively (Additional file 3). These Southern bots indicated that *WBLH3 *and *WBLH4 *respectively represented three homoeologous loci of group 1 and 3 chromosomes in common wheat.

### Expression patterns of the four *WBLH *genes revealed by RT-PCR analyses

**Figure 3:**
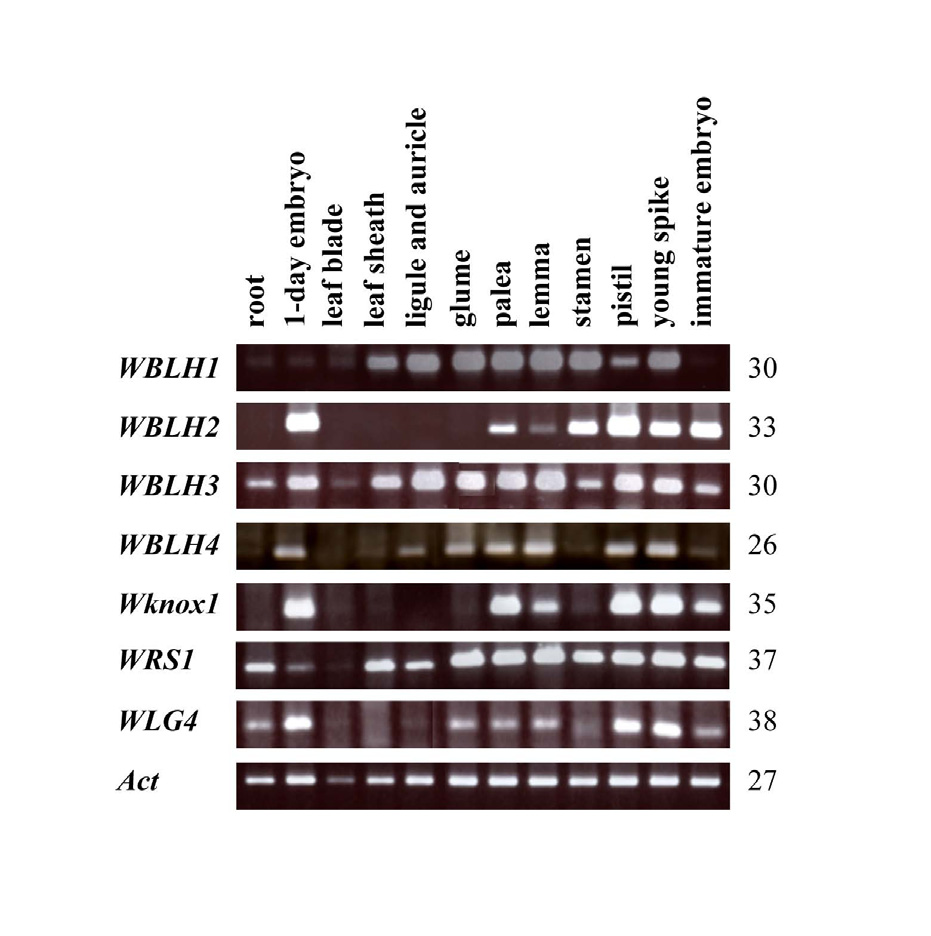
**RT-PCR analysis of four *BLH *and three *KNOX *genes in the normal line (CS)**. Total RNA was isolated from roots, embryos on day 1 of germination, leaf blades, leaf sheaths, ligules and auricle-containing leaf regions, glumes, paleae, lemmas, stamens, pistils, young spikes (15-25 mm in length) and immature embryos 10 days after anthesis. The *Act *gene was used as an internal control. Number of PCR cycles is shown on the right of each panel.

To study expression patterns of the four *WBLH *genes in wheat, RT-PCR analysis was conducting using total RNA from various tissues of CS. Single bands were clearly visualized for the four *WBLH *genes, although the gene-specific primers did not distinguish the three A, B, and D homoeoalleles of the target genes. The four wheat *BLH *genes showed tissue-specific expression patterns (Figure 3). *WBLH1 *transcripts accumulated predominantly in floral organs than in vegetative organs, suggesting that these genes play important roles in wheat reproductive organ development. *WBLH2 *transcripts were detected in shoot and inflorescence meristem-containing tissues, and in floral organs. *WBLH3 *expression was observed in most tissues examined, and the expression pattern of *WBLH4 *closely resembled that of *WBLH3*.

**Figure 4:**
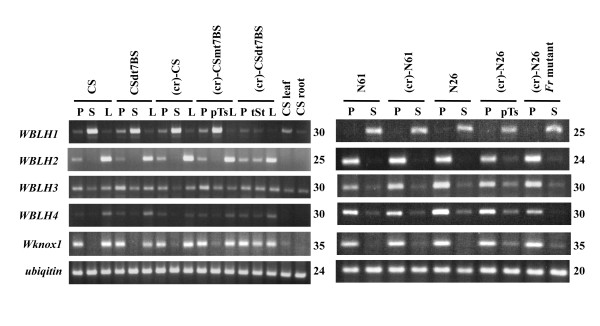
**RT-PCR analysis of *WBLH *and *Wknox1 *in various euplasmic and alloplasmic wheat lines**. The *Ubi *gene was amplified as a control for normalization. Number of PCR cycles is shown on the right of each electropherogram. P, pistil; S, stamen; L, lodicule; pTs, partially transformed stamen; tSt, transformed stamen.

**Figure 5:**
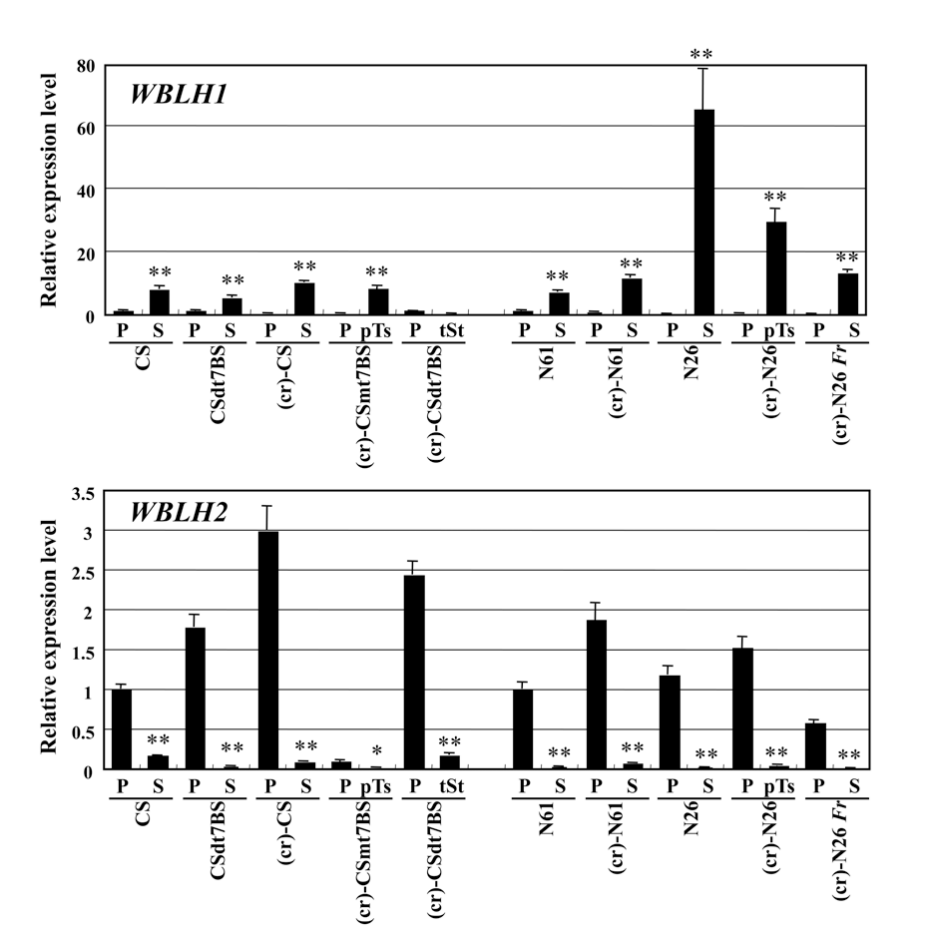
**Quantitative RT-PCR analysis of *WBLH1 *and *WBLH2 *in euplasmic and alloplasmic lines of CS**. *Act *was used as an internal control. The transcript levels are shown as values relative to the mRNA levels in pistils of CS. Data are represented as means with standard deviation (*n *= 3 or *n *= 4). One and two asterisks respectively indicate statistical significance between pistil and stamen at the 5% and 1% levels (Student's *t *test). P, pistil; S, stamen; pTs, partially transformed stamen; tSt, transformed stamen.

Next, to compare expression patterns of the four *WBLH *genes in wheat florets, semi-quantitative and quantitative RT-PCR analyses were conducted using total RNA isolated from floral organs of various alloplasmic wheat lines with *Ae. crassa *cytoplasm. *WBLH1 *transcripts were more abundantly accumulated in stamens than in pistils and lodicules of CS, and were observed at a low level in leaves of CS seedlings (Figure 4). Similarly, abundant accumulation of *WBLH1 *transcripts was observed in partially transformed stamens of (cr)-CSmt7BS, but no significant difference in the *WBLH1 *transcript level was observed between stamens of (cr)-CS and the partially transformed stamens of (cr)-CSmt7BS (Figure 5). In transformed stamens of (cr)-CSdt7BS, the transcript level was similar to that in the pistils of both euplasmic and alloplasmic CS lines. To confirm abundant expression in stamens, *WBLH1 *expression was also analyzed using the other series of euplasmic and alloplasmic wheat lines. The *WBLH1 *transcripts similarly accumulated in stamens of all the lines examined (Figure 4). In partially transformed stamens of (cr)-N26, the *WBLH1 *transcript level was significantly reduced compared with euplasmic N26 (Figure 5).

Abundant accumulation of *WBLH2 *transcripts was observed in pistils and lodicules, with no transcript detected in leaves and roots of CS (Figure 4). The abundant expression of *WBLH2 *in pistils was also observed in the other euplasmic and alloplasmic lines. The *WBLH2 *transcript levels in transformed stamens of (cr)-CSdt7BS and partially transformed stamens of (cr)-CSmt7BS and (cr)-N26 were significantly reduced compared with those in pistils, whereas transformed stamens of (cr)-CSdt7BS had an increased *WBLH2 *transcript level compared with normal stamens of CSdt7BS (Figure 5). The *WBLH2 *transcripts in the partially transformed stamens of (cr)-CSmt7BS and (cr)-N26 accumulated at lower levels than those of fully transformed stamens.

*WBLH3 *and *WBLH4 *transcripts more abundantly accumulated in pistils and lodicules compared with stamens of CS (Figure 4). *WBLH3 *expression was also detected in leaves and roots. *WBLH4 *transcript levels were more abundant in the lodicules than in the pistils. In pistils of the other euplasmic and alloplasmic lines, predominant expression of *WBLH3 *and *WBLH4 *was clearly observed. The *WBLH3 *and *WBLH4 *transcript levels in transformed stamens of (cr)-CSdt7BS were similar to those in pistils.

*Wknox1 *is an ortholog of the maize *kn1 *homeobox gene functioning mainly in shoot, inflorescence and floral meristem [40,42]. *WRS1 *and *WLG4 *are wheat orthologs of maize *rough sheath1 *(*rs1*) and *liguleless4 *(*lg4*) *KNOX *genes, respectively [43,44]. Maize *rs1 *and *lg4 *belong to the class I *KNOX *gene family and play important roles in maintenance of shoot apical meristem (SAM) indeterminancy and differentiation of lateral organs [47-49]. Transcripts of *Wknox1*, *WRS1 *and *WLG4 *accumulated abundantly in SAM-containing embryos and young spikes and in floral organs, but not in fully developed leaf blades (Figure 3). Accumulation of *Wknox1 *transcript was observed in pistils and lodicules of CS (Figure 4). Low levels of *Wknox1 *transcripts were detected in stamens of euplasmic and alloplasmic lines of CS, whereas slight *Wknox1 *transcript accumulated in partially transformed stamens of (cr)-CSmt7BS. *Wknox1 *transcripts accumulated abundantly in transformed stamens of (cr)-CSdt7BS, and the accumulation level was similar to that in pistils. Abundant expression of *Wknox1 *in pistils was also observed in the other euplasmic and alloplasmic lines.

### In situ localization of the four *WBLH *transcripts in pistils

**Figure 6:**
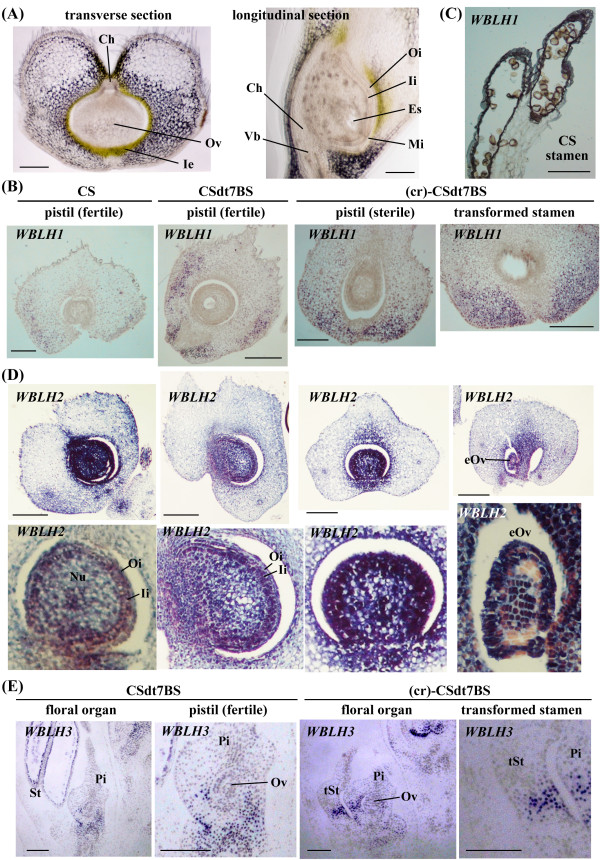
**In situ hybridization of *WBLH *transcripts in floral organs of CS (wild-type), CSdt7BS (normal flower) and (cr)-CSdt7BS (flower showing pistillody)**. (A) Transverse (left) and longitudinal (right) sections of a normal, fully developed pistil from CSdt7BS. The ovule is enveloped by an inner epidermis. The vascular bundle is connected to the chalaza. (B) In situ localization of *WBLH1 *transcripts in transverse sections of pistils of euplasmic and alloplasmic CS lines. (C) In situ localization of *WBLH1 *transcripts in longitudinal sections of the CS stamens. (D) In situ localization of *WBLH2 *transcripts in transverse sections of pistils and stamens of euplasmic and alloplasmic CS lines. Lower panels show higher magnification images of the pistils. The magnified image of CS was derived from another section for in situ hybridization analysis of *WBLH2 *transcript. (E) In situ localization of *WBLH3 *transcripts in longitudinal sections of developing florets from euplasmic and alloplasmic lines of CSdt7BS. St, stamen; Pi, pistil; tSt, transformed stamen; Ov, ovule; Ie, inner epidermis; Ch, chalaza; Vb, vascular bundle; Es, embryo sac; Mi, micropyle; Oi, outer integument; Ii, inner integument; Nu, nucellus; eOv, ectopic ovule. Scale bars represent 100 μm.

Fully developed ovules in normal pistils are surrounded by the inner and outer integuments, all enveloped by an inner epidermis (Figure 6A) [23]. To compare the expression patterns of the four *WBLH *genes in pistil development of common wheat in detail, in situ hybridization analysis was conducted using pistils and young spikes at the floral organ developing stage, in which stamen and pistil primordia develop. In transverse sections of pistils from CS and CSdt7BS, accumulation of *WBLH1 *transcript was found in the ovary, but not the ovule (Figure 6B). In both pistils and transformed stamens of (cr)-CSdt7BS, *WBLH1 *transcripts were localized to the ovary but not the ovule. No significant differences in *WBLH1 *localization were observed among fertile pistils, sterile pistils, and transformed stamens. *WBLH1 *transcripts were also found in longitudinal sections of anthers, especially in tapetum and anther epidermis of CS (Figure 6C).

*WBLH2 *transcripts highly accumulated at the stage of floral organ development in the ovule of the CS, CSdt7BS and (cr)-CSdt7BS pistils (Figure 6D). *WBLH2 *mRNA was present at higher levels in the inner integument than in the outer integument and nucellus of the pistils. No significant difference in *WBLH2 *mRNA localization was observed between fertile and sterile pistils. Even in ectopic ovules of the transformed stamens, *WBLH2 *transcripts highly accumulated, although the integument developed incompletely.

In longitudinal sections of young spikes at the floral organ developing stage from CSdt7BS, *WBLH3 *mRNA was detected in the anther walls of the stamen and the basal region of the carpel but not in the ovule (Figure 6E). *WBLH3 *transcripts accumulated in the central region of the transformed stamens of (cr)-CSdt7BS, where the ectopic ovule was presumed to develop. Similar expression patterns were observed for *WBLH4 *(data not shown).

### Protein-protein interaction between wheat BLH and KNOX

**Figure 7:**
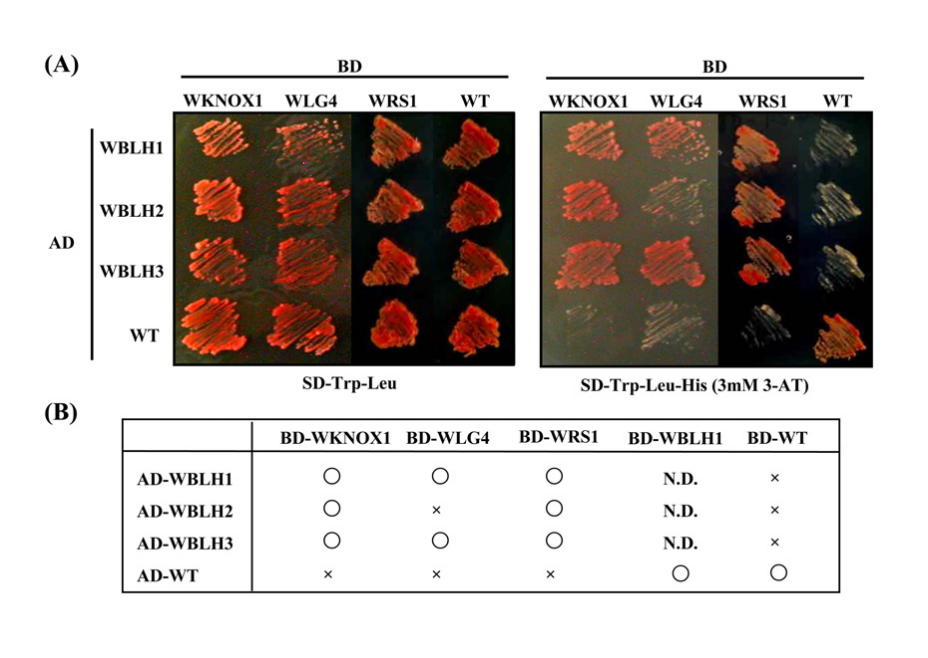
**Interaction of WBLH and WKNOX proteins in a yeast two-hybrid system**. (A) Transformed yeast lines were grown on SD medium without Trp and Leu (SD-Trp-Leu) (left) or SD medium lacking Trp, Leu and His (SD-Trp-Leu-His) (right). Protein interaction is assessed by the viability of yeast transformants on SD-Leu-Trp-His containing 3mM 3-AT. AD-WT and BD-WT, control plasmids. (B) Summary of the results of the yeast two-hybrid assay. Circles and crosses respectively indicate interacting or activating, and noninteracting or nonactivating. N.D., not determined. Interaction of BD-WBLH1 and AD-WT is not shown.

Protein-protein interactions between BLH and KNOX were previously reported in *Arabidopsis *and barley [31,34]. The BLH-KNOX heterodimers play important roles in plant development [30,32,33,50]. To confirm the interactions between wheat BLH and KNOX proteins, a yeast two-hybrid assay was conducted. Entire ORF sequences of *Wknox1*, *WRS1 *and *WLG4 *were fused to the nucleotide sequence for the yeast GAL4 DNA binding domain (BD-WKNOX1, BD-WRS1 and BD-WLG4), and the *WBLH1*, *WBLH2 *and *WBLH3 *ORFs were fused to the sequence for the yeast GAL4 transcriptional activation domain (AD-WBLH1, AD-WBLH2 and AD-WBLH3). The yeast two-hybrid assay indicated that WBLH1 interacts with WKNOX1, WRS1 and WLG4 (Figure 7). WBLH3 also showed interaction with the three wheat KNOX proteins. WBLH2, however, formed a complex with WKNOX1 and WRS1, but not with WLG4.

**Figure 8:**
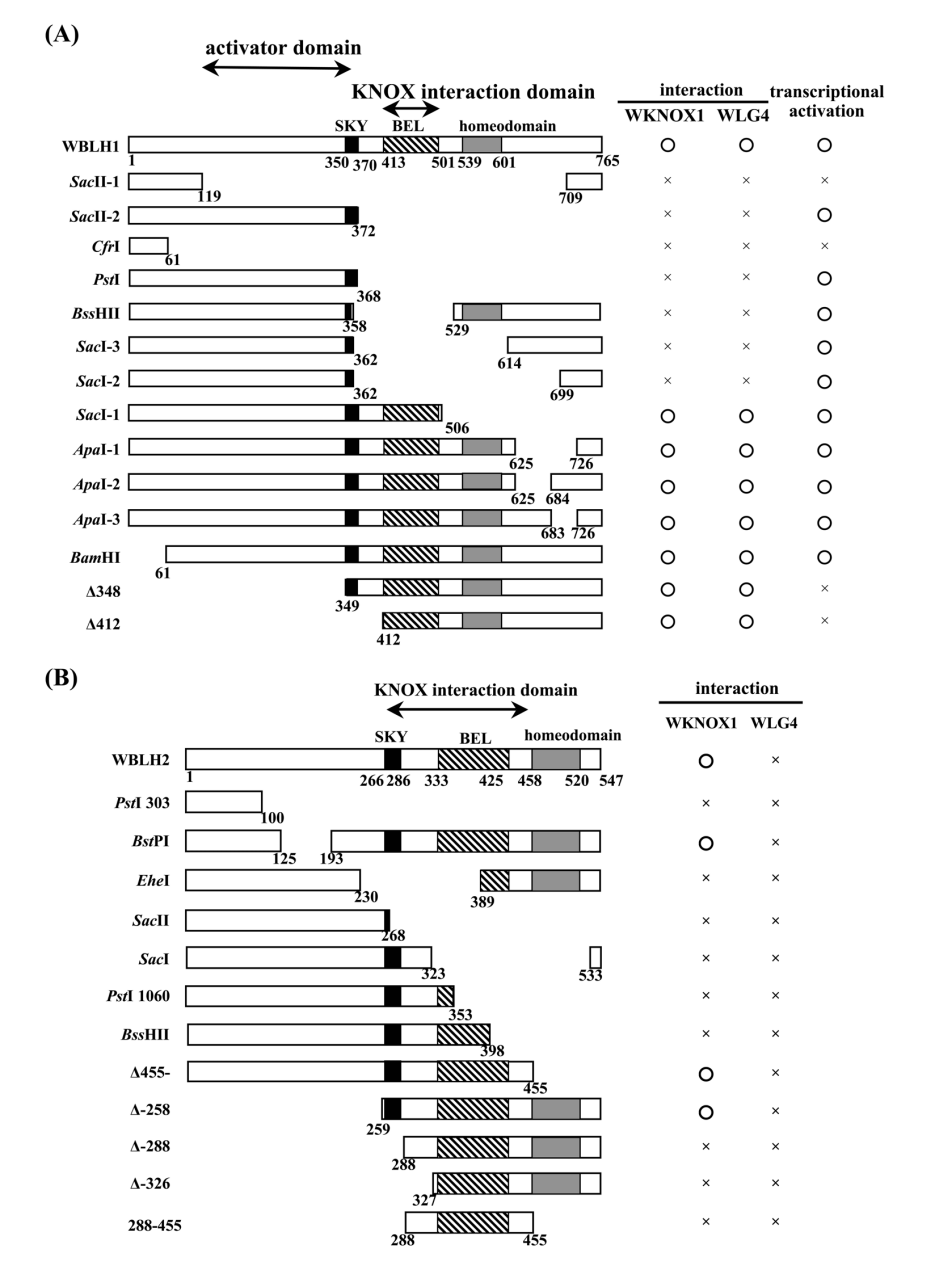
**Structures of deletion derivatives of WBLH1 and WBLH2 and their interactions with wheat KNOX proteins**. The SKY, BEL and homeobox domains are shown by yellow, green and red boxes, respectively. Numbers indicate amino acid residue numbers at the deletion break points. Interaction activity with WKNOX1 and WLG4 and transcriptional activity are also summarized on the right side. Circles indicate interacting or activating, and crosses indicate noninteracting or nonactivating.

To determine the KNOX-interacting domain of wheat BLH proteins, deletion-mutant constructs of WBLH1 and WBLH2 were produced, and protein-protein interaction of the WBLH1 and WBLH2 deletion proteins with WKNOX1 and WLG4 was examined by yeast two-hybrid assay (Figure 8). NoWBLH1 deletion construct lacking the BEL domain showed positive interaction with WKNOX1 and WLG4 (Figure 8A). Neither deletion in the SKY domain nor the homeodomain of WBLH1 had any effect on protein-protein interactions with the two KNOX proteins. The N-terminal region of WBLH1 was also not required for the interactions. *Arabidopsis *BEL1 interacts with KNOX proteins through the N-terminal to homeodomain regions [34]. The interaction domain with KNOX was more restricted in WBLH1 than in BEL1. Similarly, deletions in the SKY and BEL domain region of WBLH2 abolished the interaction with WKNOX1, and a WBLH2 deletion without the homeodomain had no influence on the interaction (Figure 8B). Deletion analysis of WBLH2 indicated that the region from the SKY domain to the BEL domain was required for the interaction with WKNOX1. In addition, no deletion derivatives of WBLH2 showed positive interactions with WLG4, meaning the absence of any region inhibiting the interaction with WLG4 in WBLH2.

The entire ORF of *WBLH1 *was fused to the nucleotide sequence for the yeast GAL4 DNA binding domain. Yeast transformants carrying the BD-WBLH1 construct survived on His^- ^selection medium, indicating that WBLH1 could activate transcription of the reporter gene used in yeast. To identify the activation-related domain, deletion derivatives of WBLH1 fused to the DNA binding domain were introduced, and transformants were recovered on His^- ^selection medium. No WBLH1 constructs lacking either the SKY, BEL or homeodomains lost function as a transcriptional activator (Figure 8A). Deletion of the N-terminal region failed to activate reporter gene expression. These results revealed that the N-terminal region (119th to 349th amino acid residues) was essential for transcriptional activation.

## Discussion

### *WBLH2 *function is distinct from those of other three *BLH *genes

*Bell1*-type homeobox genes play important roles in plant development. In this study, four wheat *BLH *genes were isolated and named *WBLH1 *to *WBLH4*. WBLH1, a putative ortholog of barley JuBEL1 [31], was highly similar to *Arabidopsis *SAWTOOTH1 (SAW1) and SAWTOOTH2 (SAW2), which act redundantly to suppress expression of the *KNOX *gene *BREVIPEDICELLUS *(*BP*) in leaf margins [30]. *SAW1 *and *SAW2 *expression was also observed in lateral organs including the adaxial side of developing sepals but not in developing ovules, and therefore *SAW1 *and *SAW2 *are unlikely to be redundant with *BEL1 *function in ovule development, although the two genes are the most closely related in sequence to *BEL1 *[30]. *WBLH1 *was expressed in the ovary but not the ovule (Figure 6B), indicating that *WBLH1 *function also does not correspond to that of *BEL1*.

Both BEL1 and SAW1 are able to interact with class I KNOX proteins, STM, BP and KNAT2, and with the class II KNOX protein KNAT5, but not with other class II KNOX proteins, KNAT3, KNAT4 and KNAT7 [30,35]. The class I *KNOX *genes are considered to play a role in establishment and maintenance of meristematic identity and in the initiation of lateral organ primordia, and their expression patterns were temporally and spatially regulated, whereas expression of the class II *KNOX *genes is constitutive [47,51,52]. Similarly, WBLH1 could interact with three class I KNOX proteins, WKNOX1, WRS1 and WLG4 (Figure 7). *Wknox1*, *WRS1 *and *WLG4 *are related to *KNAT1*, *BP *and *KNAT5*, respectively [36,40,43]. Barley JuBEL1 also interacts with class I KNOX proteins BKN1 and BKN3, but not with class II KNOX protein BKN7 [31]. The interacting domain of WBLH1 could be defined to the BEL domain in this study (Figure 8), which was included in the interacting region of JuBEL1 [31]. These results imply evolutionary conservation of the protein-protein interaction between the class I KNOX proteins and WBLH1-related proteins since the divergence of monocotyledonous and dicotyledonous plants. In animals, interaction between two types of TALE-homeobox protein families plays important roles in development, and an N-terminal portion of the PBX subclass proteins is essential for heterodimerization with the MEINOX subclass proteins [53,54]. The MEINOX-PBX complex in animals is evolutionarily conserved as the KNOX-BLH complex in plants [34]. KNOX-BLH interaction likely alters intracellular localization of the KNOX transcription factor [55], and the KN1-BLH complex binds to the specific DNA motif, TGACAG(G/C)T, with higher affinity compared with KN1 alone [56]. These observations surely indicate the significance of KNOX-BLH complex formation in various plant developmental processes.

WBLH3 also interacted with the three class I KNOX proteins (Figure 7). *WBLH3 *was phylogenetically close to *WBLH4*, a putative ortholog of barley *JuBEL2 *[31,57], although the chromosomal locations of *WBLH3 *and *WBLH4 *were different. RT-PCR and in situ mRNA hybridization analyses revealed that the gene expression profile of *WBLH3 *was generally identical to that of *WBLH4*. *WBLH3 *and *WBLH4 *were homologous to *Arabidopsis BLH1 *(Figure 2B). *WBLH3 *and *WBLH4 *expression was observed in the basal region of the carpel but not the ovule (Figure 6E). Similarly, *Arabidopsis BLH1 *expression is restricted to the transmitting tract and the base of the funiculus, but is not observed in the ovule or embryo sac [58]. Therefore, the *WBLH3*/*4 *expression patterns were well conserved with that of *BLH1*. Misexpression of *BLH1 *affects normal embryo sac development in ovules of the *Arabidopsis eostre *mutant [58]. A mutation of the class II KNOX gene *KNAT3 *suppresses the *eostre *mutant phenotype [58], and KNAT3 is able to interact with BLH1 [58]. Hackbusch et al. [59] and Pagnussat et al. [58] also reported that the function of the *Arabidopsis *KNOX-BLH complexes requires the ovate family proteins. No misexpression of *WBLH3*/*4 *was observed in ovules of the euplasmic and alloplasmic wheat lines, and therefore we have no information about functional orthology between *BLH1 *and *WBLH3*/*4*. WBLH3 displays similarity to the rice qSH1 homeobox protein, a putative ortholog of the *Arabidopsis *REPLUMLESS (RPL) homeobox protein [29,60]. *RPL *and *qSH1 *are associated with seed shattering. *RPL *is expressed in *Arabidopsis *stem, pedicels and replum of developing ovaries, and *qSH1 *is expressed in rice inflorescence meristem, anther and the boundary of the spikelets. *WBLH3 *might function to define the boundary regions as do *RPL *and *qSH1*.

*WBLH2*, closely related to the *Arabidopsis ATH1 *homeobox gene, was phylogenetically distinct from other wheat *BLH *genes (Figure 2B). *ATH1 *was originally isolated as a light-induced homeobox gene [46]. *ATH1 *is expressed in shoot, inflorescence and floral meristems, developing stamens and carpels, and basal regions of lateral organs including leaves, sepals and petals [61]. The mutant phenotype of *ath1 *indicates that *ATH1 *controls the development of the boundary region between shoot lateral organs and the stem [61]. The ATH1-STM complex is associated with initiation and maintenance of *Arabidopsis *SAM [50]. *AHT1 *expression in stamens and carpels is consistent with the activation of *ATH1 *by *AGAMOUS *[62]. In contrast to the *ATH1 *expression pattern, *WBLH2 *was abundantly expressed in ovules but not in ligule or auricle regions (Figure 3, Figure 6D). The *WBLH2 *expression pattern is likely to be analogous to that of *BEL1 *rather than *ATH1*. Especially in integuments of ovules, *WBLH2 *transcripts accumulated abundantly, indicating that *WBLH2 *might be associated with development of integuments in wheat. The ovule-specific expression of *WBLH2 *clearly differed from the expression patterns of three other wheat *BLH *genes. In addition, of the class I KNOX proteins examined, WBLH2 was not able to interact with WLG4 (Figure 7). The interacting region of WBLH2 with WKNOX1 was defined from the SKY domain to the BEL domain, which was broader than the interacting region of WBLH1 with WKNOX1 and WLG4 (Figure 8). These observations reveal that the functional features of *WBLH2 *are distinct from those of the other *BLH *gene functions in wheat development. In particular, the role of *WBLH2 *in ovule development should be elucidated in further studies.

### Altered expression of *BEL1*-type homeobox genes in floral organs of alloplasmic lines

Pistillody is one of the typical phenomena appearing in nuclear-cytoplasm incompatibility of higher plants. For expression of pistillody, the expression patterns of a large number of nuclear genes including class B MADS-box genes are altered [6,63]. The downregulation of the class B MADS-box genes in stamen primordia results in homeotic conversion at floral whorl three in the pistillody-showing alloplasmic lines as well as loss-of-function mutants of the class B MADS-box genes. Within their pistil-like stamens, ectopic ovules are surely formed as reported in *Arabidopsis*, rice and alloplasmic wheat [6,64,65]. Our previous studies revealed that wheat class B MADS-box genes *WPI1*, *WPI2 *and *WAP3 *are downregulated at floral whorl three in alloplasmic wheat lines showing pistillody [6,14], whereas two class C MADS-box genes, *WAG-1 *and *WAG-2*, and *TaDL*, an ortholog of rice *DROOPING LEAF *(*DL*), are ectopically expressed in primordia of the pistil-like transformed stamens [15,16,23,66]. After floral organ identity is homeotically changed at whorl three, ovule formation is likely to automatically occur within the transformed stamens through expression of the class D MADS-box gene *TaAGL2*/*WSTK *[23]. During ectopic ovule development, expression of *WBsis *and *WANT-1 *is observed in integuments of the ovules [16,23]. *WBLH2 *was also strongly expressed in integuments not only of normal ovules in pistils but also of ectopic ovules in transformed stamens (Figure 6). *WBLH1*/*WBLH3*/*WBLH4 *expression was observed in the basal boundary region of the ovary in both normal pistils and transformed stamens. These observations suggest that expression of the four *WBLH *genes is associated with development of transformed stamens in alloplasmic wheat lines with *Ae. crassa *cytoplasm.

Ectopic ovules in transformed stamens and ovules in pistils of (cr)-CSdt7BS are sterile due to abnormal development of the inner epidermis and integuments [6,23]. It was thought that aberrant expression of *WBsis *and a reduced transcript level of *WANT-1 *might at least partially result in the sterility of the ovules and ectopic ovules [16,23] (Murai et al. unpublished results). Such an obvious alteration of gene expression in the ovules and ectopic ovules was not observed in *WBLH2*. The *WBLH2 *expression pattern in the sterile pistils seemed to be identical to that in normal ovules of fertile pistils (Figure 6D). Therefore, we have no evidence for *WBLH2 *being associated with the female sterility of the transformed stamens and pistils in (cr)-CSdt7BS. The regulatory pathway of *WBLH2 *expression in ovule development might be independent of those of *WBsis *and *WANT-1*. After *WAG-1 *and *WAG-2 *expression in the transformed stamens and sterile pistils, *WSTK *initiates ovule development [23]. *WBLH2 *expression occurs continuously in the developing ovules of the transformed stamens and sterile pistils, whereas *WBsis *is ectopically expressed in the marginal region of ovule primordia and *WANT-1 *expression level is reduced around ovule primordia [16,23] (Murai et al. unpublished results). After ovule initiation via *WSTK*, some of the ovule development-related genes such as *WBsis *and *WANT-1 *should be abnormally expressed, which in turn might be associated with the aberrant ovule development. In further studies, signal cascades from initiation of ovule primordia to *WBsis*/*WANT-1 *or *WBLH2 *expression should be elucidated to investigate how the *Ae. crassa *cytoplasm and *Rfd1 *affect female sterility in the pistillody-exhibiting alloplasmic wheat lines.

## Conclusions

Wheat *BLH *genes consist of a small multigene family. Four wheat *Bell1*-like homeobox (*BLH*) genes, *WBLH1 *to *WBLH4*, were isolated in this study. A yeast two-hybrid assay showed that KNOX-BLH interaction was conserved in wheat similarly to in various other plant species, indicating the significance of KNOX-BLH complex formation in wheat developments. Of the class I KNOX proteins examined, WBLH2 was unable to interact with WLG4. The interacting region of WBLH2 with WKNOX1 was defined from the SKY domain to the BEL domain, which was broader than the interacting region of WBLH1 with WKNOX1 and WLG4. In addition, the ovule-specific expression of *WBLH2 *clearly differed from the expression patterns of three other wheat *BLH *genes. Therefore, the functional features of *WBLH2 *are likely to be distinct from other *BLH *gene functions in wheat development. *WBLH2 *was also strongly expressed in integuments not only of normal ovules in pistils but also of ectopic ovules in transformed stamens. *WBLH1*/*WBLH3*/*WBLH4 *expression was observed in the basal boundary region of the ovary in both normal pistils and transformed stamens. These results indicated that the four *WBLH *genes may participate in development of pistils and transformed stamens but are not associated with female sterility in alloplasmic wheat lines with *Ae. crassa *cytoplasm.

## Authors' contributions

KMi designed the experiments, carried out the molecular genetic studies, and drafted the manuscript. HH participated in the real-time RT-PCR analysis. CH carried out the in situ hybridization. KMu participated in the design and coordination of the study and helped to draft the manuscript. ST conceived of the study, designed and coordinated the study, and wrote the manuscript. All authors read and approved the final manuscript.

## Supplementary Material

Additional file 1 **Comparison of nucleotide sequences of 63 RT-PCR products with BLH-degenerate primers**. Click here for file

Additional file 2 **Amino acid sequence alignment of three conserved domains in the WBLH proteins**. Click here for file

Additional file 3 **Copy number and chromosome assignment of the four *WBLH *genes**. Click here for file
