# Supplementary material for: Characterization of wheat Bell1-type homeobox genes in floral organs of alloplasmic lines with Aegilops crassa cytoplasm
Source: BMC Plant Biol. 2011 Jan 4;11:2. doi: 10.1186/1471-2229-11-2 (PMC3022553; doi:10.1186/1471-2229-11-2)
Supplement: Additional file 1 — Comparison of nucleotide sequences of 63 RT-PCR products with BLH-degenerate primers. [file 1471-2229-11-2-S1.PDF]

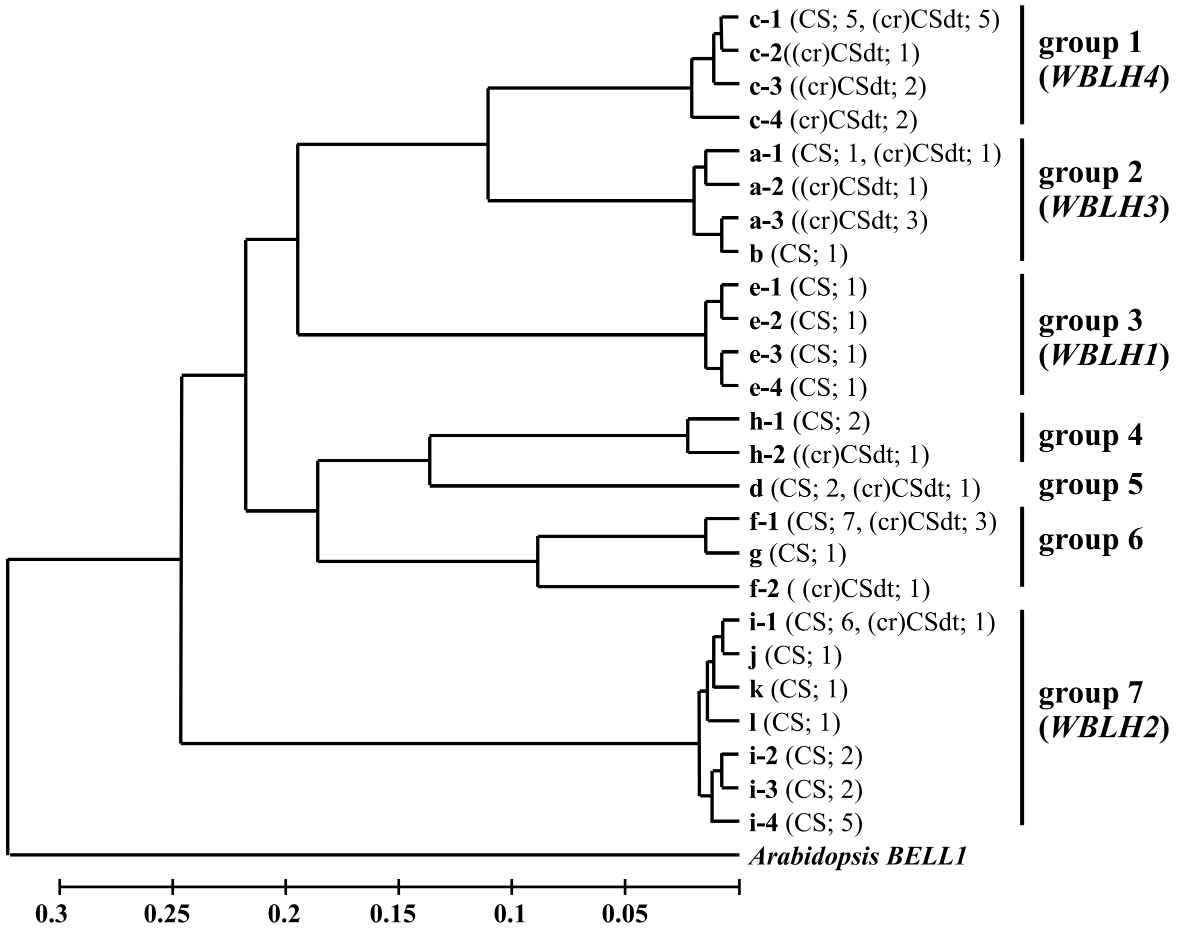

Additional file 1. Comparison of nucleotide sequences of 63 RT-PCR products with BLH-degenerate primers.

A phylogenetic tree based on the nucleotide sequences was constructed according to the UPGMA method. Clone numbers with identical sequences are shown in parentheses together with their derived wheat lines, CS and (cr)-CSdt7BS ((cr)CSdt). The 63 sequences were divided into 12 families (a to l) based on their polymorphisms, and clustered into seven phylogenetically distinct groups (group 1 to group 7).
