# Supplementary material for: Characterization of wheat Bell1-type homeobox genes in floral organs of alloplasmic lines with Aegilops crassa cytoplasm
Source: BMC Plant Biol. 2011 Jan 4;11:2. doi: 10.1186/1471-2229-11-2 (PMC3022553; doi:10.1186/1471-2229-11-2)
Supplement: Additional file 2 — Amino acid sequence alignment of three conserved domains in the WBLH proteins. [file 1471-2229-11-2-S2.PDF]

(A) SKY domain

```
WBLH1    1 LRNSKYTRAA QELLDEFCSV G 21
JUBEL1   1 ********** ********** * 21
WBLH2    1 *PR*G*AHI* ****NG*AGC L 21
WBLH3    1 *GR*RFLGP* *K**E*I*D* * 21
WBLH4    1 *GR*RFLGP* *K**E*I*D* * 21
JUBEL2   1 *GR**FLVP* *R**E*I*D* * 21
BEL1     1 IGS***LSP* ****S****L * 21
```

(B) BEL domain

```
WBLH1    1 PQLSPADRFE QQRKKAKLIS MLDEVDRRYN HYCDQMQMVV NFFDSVM---  50
JUBEL1   1 ********** ********** ********** ********** *******---  50
WBLH2    1 HSADGGRWME A**VRND*LK LLQLMDQRC* RCF*DI**TA SK*S*MVAHP  50
WBLH3    1 AEGVTVSGA* **WR*TR*** LME**CK**R Q*YQ*L*A*I SS*ET*A---  50
WBLH4    1 DTSG*MSGA* **W**TR*** *ME**CK**R Q*YQ*V*A*I AS*E**A---  50
JUBEL2   1 DAAG*MYGA* **W**TR*** *ME**CK**R Q*YQ*V*SAI AS*ET*A---  50
BEL1     1 *P*HSLEFM* L*KR****L* **E*LK***G **RE**RVAA AA*EAAV---  50

WBLH1    51 -GFGAATPYT ALAQKAMSRH FRCLKDAIAA QLRHTCELLG EKD  93
JUBEL1   51 -********* ********** ********** ********** ***  93
WBLH2    51 G*G*G*IAPP PF**C*L*AV Y*R*RKR*TG LIVAVAQRS* GHG  93
WBLH3    51 -*LSN*A*FA SI*LRT**K* *KY**ST*QS ***N*SKVAA G**  93
WBLH4    51 -**SN*A*F* ***LRV*A** ***I*GM*LS ***N*SKMPV -*E  93
JUBEL2   51 -**SN*A*F* ***LRV*AK* *KTI*EM*LS ***N*SKMPV -*G  93
BEL1     51 -*L*G*EI** ***SR***** ******GLVG *IQA*SQA** *RE  93
```

(C) homeodomain

```
WBLH1    1 AWRPQRGLPE RSVSILRSWL FEHFLHPYPS DADKHLLARQ TGLSRNQVSN WFINARVRLW KPM 63
JUBEL1   1 ********** ********** ********** ********** ********** ********** *** 63
WBLH2    1 S********* K**AV*KA*M **N**R***K *HE*DM**AR S****S**** ********** *** 63
WBLH3    1 I********* *A**V**A** *********T *S**QM**K* ***T****** ********** *** 63
WBLH4    1 I********* ***TV**A** *********T *G**QM**K* ***T****** ********** *** 63
JUBEL2   1 I********* ***TV**A** *********T *G**QM**K* ***T****** ********** *** 63
BEL1     1 P********* *A*TT**A** ********** *V***I**** *****S**** ********** *** 63
```

Additional file 2. Amino acid sequence alignment of three conserved domains in the WBLH proteins.

In addition to the four WBLH proteins, barley JUBEL1 and JUBEL2, *Arabidopsis* BEL1 were aligned. Homeodomains were highly conserved among the BLH proteins (C), whereas SKY (A) and BEL (B) domains were less conserved than homeodomains.
