# Supplementary material for: Characterization of wheat Bell1-type homeobox genes in floral organs of alloplasmic lines with Aegilops crassa cytoplasm
Source: BMC Plant Biol. 2011 Jan 4;11:2. doi: 10.1186/1471-2229-11-2 (PMC3022553; doi:10.1186/1471-2229-11-2)
Supplement: Additional file 3 — Copy number and chromosome assignment of the four WBLH genes. [file 1471-2229-11-2-S3.PDF]

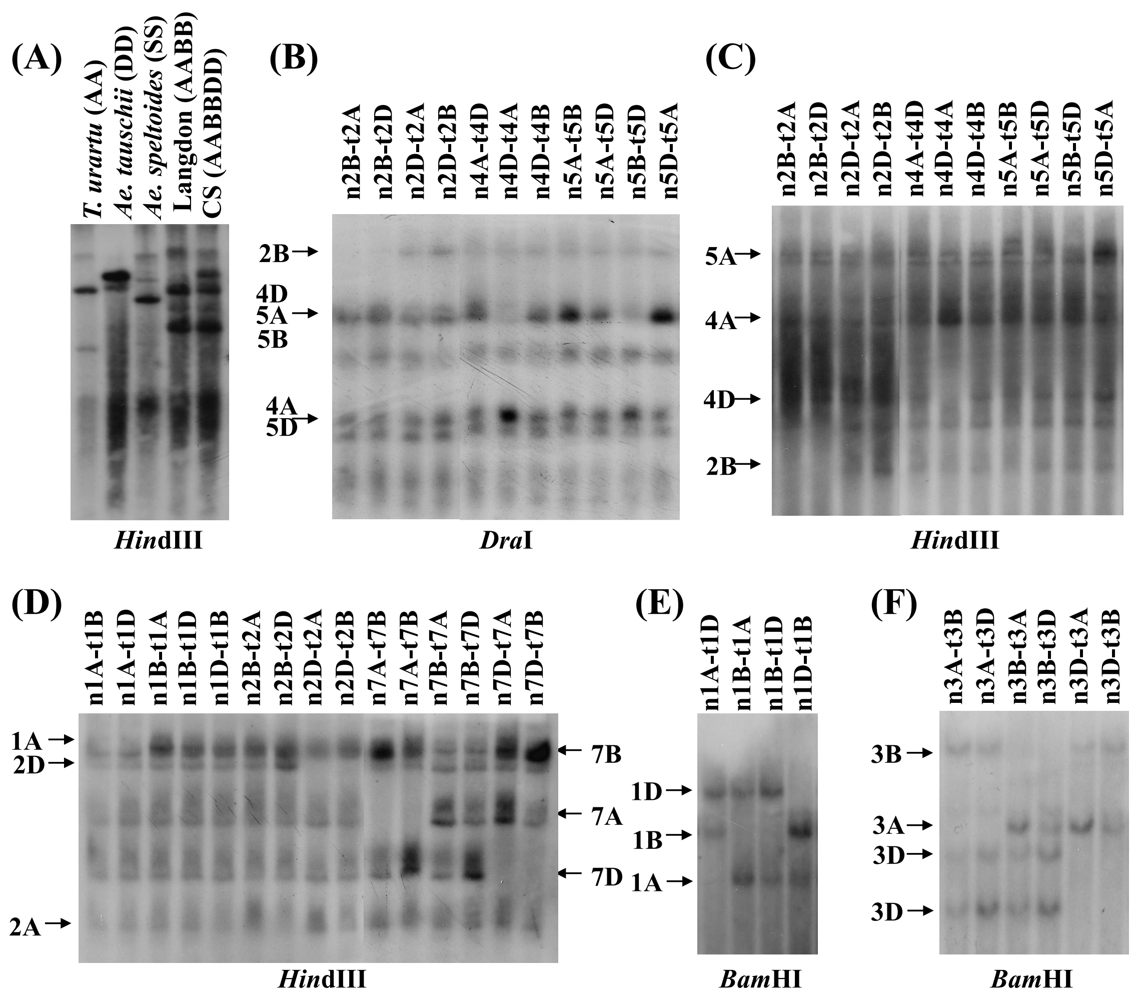

Additional file 3. Copy number and chromosome assignment of the four *WBLH* genes.

(A) Southern blot analysis of *WBLH1*. Total DNA was digested with *Hin*dIII. (B, C) Nulli-tetrasomic analysis of *WBLH1.* Total DNA samples from a nulli-tetrasomic series of CS were digested with *Dra*I (B) and *Hin*dIII (C). (D, E, F) Nulli-tetrasomic analysis of *WBLH2* (D)*, WBLH3* (E) and *WBLH4* (F). Total DNA was digested with *Hin*dIII (D) and *Bam*HI (E, F). Probe positions are shown in Fig. 2A. n, nullisomic; t, tetrasomic.
